# A Review of Wood Polymer Composites Rheology and Its Implications for Processing

**DOI:** 10.3390/polym12102304

**Published:** 2020-10-08

**Authors:** Valentina Mazzanti, Francesco Mollica

**Affiliations:** Department of Engineering, University of Ferrara, 44122 Ferrara, Italy; francesco.mollica@unife.it

**Keywords:** wood plastic composites, natural fibers, rheology, processing, viscosity, non-Newtonian fluids, compounding, material formulation

## Abstract

Despite the fact that wood polymer composites are interesting materials for many different reasons, they are quite difficult to shape through standard polymer processing techniques, such as extrusion or injection molding. Rheological characterization can be very helpful for understanding the role played by the many variables that are involved in manufacturing and to achieve a good quality final product through an optimized mix of formulation and processing parameters. The main methods that have been used for the rheological characterization of these materials are capillary and parallel plate rheometry. Both are very useful: rotational rheometry is particularly convenient to investigate the compounding phase and obtain structural information on the material, while capillary viscometry is well suited to understand final manufacturing. The results available in the literature at the moment are indeed very interesting and are mostly aimed at investigating the influence of the material formulation, the additives in particular, on the structural, mechanical, and morphological properties of the composite: despite a good number of papers, though, it is difficult to draw general conclusions, as many issues are still debated. The purpose of this article was to overview the state of the art and to highlight the issues that deserve further investigation.

## 1. Introduction

Wood polymer composites, also known as wood plastic composites or WPC, are thermoplastic polymers that contain a certain amount of wood in the form of flour or short fibers [1,2]. Despite the fact that they can be easily processed through typical woodworking procedures, they can also be manufactured like ordinary plastics, e.g., by extrusion or injection molding [3], and formed through additive manufacturing techniques, such as fused deposition modeling (FDM) [4,5].

They are normally used as a substitute for natural wood (e.g., in fencing, flooring and decking) and their usage is convenient in wet working environments or anyway in close contact with water: the hydrophobic polymer isolates and protects the hydrophilic wood fibers, and this in turn increases durability and requires less maintenance interventions, at least up to some extent [6,7]. Noteworthy are also their applications in acoustics [8,9,10] and in the automotive field [11,12].

The main advantages of wood as a filler are cost reduction and an improvement in the environmental characteristics of the resulting composite. The former is quite obvious, as wood particles are usually low-cost materials, often coming from agricultural or industrial waste. The latter is also clear, since a relevant fraction, sometimes as high as 60 wt.% or 70 wt.% of a non-biodegradable material of fossil origin is being substituted by a more ecofriendly constituent [13]. An even greater environmental advantage can be obtained if the polymer is also a biodegradable plastic of natural origin [14,15,16,17,18,19].

Natural fibers as fillers, though, have their drawbacks. First of all, the choice of the matrix is quite limited. In fact, the natural fiber constituents, i.e., lignin, hemicellulose, and cellulose, degrade in the presence of oxygen at 100, 195, and 250 °C, respectively [20]; therefore, it is accepted that only plastics that possess a melting point that is below 195 °C are a feasible choice. As a matter of fact, the only candidates are the so-called commodities, i.e., polyethylene (PE) in its high density (HDPE) and low density (LDPE) variants, polypropylene (PP), poly-(vinyl chloride) (PVC), polystyrene (PS), and its copolymers, such as styrene-acrylonitrile (SAN) and acrylonitrile-styrene acrylate (ASA). In addition, some biodegradable plastics can be used as matrix, such as poly(lactic acid) (PLA) and an additional polymer that has been used is thermoplastic polyurethane (TPU). Of course, a wider choice for the polymeric matrix would be possible if cellulose or nanocellulose are used in substitution of natural fibers [21,22,23,24,25,26,27].

Concerning the mechanical properties of the composite, stiffness and strength are satisfactory only if costly coupling agents are added into the material formulation: these additives are necessary for improving compatibility between the wood flour and the polymer, which otherwise would not share any relevant chemical similarities [28,29]. This aims at creating an effective load transferring interface between the natural fibers and the surrounding matrix [17]. Moreover, since WPCs are usually brittle, their formulation often contains toughening agents [30,31,32], such as styrene butadiene rubber (SBR), ethylene propylene diene monomer rubber (EPDM), or thermoplastic elastomers (TPE).

Processing is a further source of complexity. In order to gain as much as possible from the aforementioned advantages, a relatively high wood flour content, such as 50 wt.% or more, is desirable. On the other hand, high amounts of filler do increase the viscosity of the polymer melt considerably, thus making flow and, hence, forming procedures rather difficult. This problem is again tackled by compounding with suitable additives, like lubricants that promote wall slip and hence lower the energy requirements for manufacturing. Notice, indeed, that the usual strategy of decreasing melt viscosity by increasing temperature is not a viable option, due to thermal degradation. Common lubricants are stearates (e.g., zinc stearate), thermoplastic silicones (TPS), and esters or ethylene bis(stearamide) (EBSA).

For correctly designing the processing machineries, a careful rheological characterization of the molten WPC is necessary. This is not completely straightforward, primarily because of the non-Newtonian nature of these fluids. Despite a good number of scientific papers, an exhaustive review is still missing in the literature, and this paper aimed at covering these aspects. In the next section, we will outline the theory behind the main rheological characterization methods that are used, before presenting the results of the scientific literature in Section 3 and Section 4. In Section 5, we will draw the conclusions and suggest a few future lines of development.

## 2. Theoretical Background

As is well known, the viscosity η of a certain fluid is defined as the ratio between the shear stress τ and the shear rate $\dot{\gamma }$:

(1) $$\eta =\frac{\tau }{\dot{\gamma }}.$$

In the scientific literature, the viscosity of molten WPC has been measured mainly using two methods, i.e., capillary viscometry and parallel plate rheometry. In the first one, a fluid is forced by an externally applied pressure gradient through a very narrow tube of circular or rectangular uniform cross section. Strictly speaking, one talks about capillary viscometry only when circular tubes are used, while rectangular ones are actually named “slits” [33,34,35,36,37]. In the present paper, we will treat these two different geometries together, referring to them as “pressure-driven viscometers”. Following the second method, a thin layer of fluid is bounded by two circular plates and subjected to a torsional flow through the movement of one of the plates, the other one remaining fixed. Variations of this second method are also important, e.g., one of the two plates can be substituted by a cone. In this paper, we will generically call these instruments “rotational rheometers”. We will now briefly overview both methodologies.

### 2.1. Pressure-Driven Viscometry

When a fluid flows through a tube of length L, the shear stress is not uniform, but its value at the wall, $\tau_{w}$, can be easily written as a function of the pressure at the entrance of the tube P. The expressions of $\tau_{w}$ for a capillary tube of radius R and for a rectangular slit of width W and depth H are reported in Table 1.

In order to calculate viscosity, then, the shear rate at the wall ${\dot{\gamma }}_{w}$ is necessary. If the fluid is Newtonian, it can be expressed as a function of the volumetric flow rate Q, as reported in Table 1, again for the case of the capillary and the rectangular slit.

If the fluid is non-Newtonian (e.g., shear thinning), the formulae in Table 1 for the wall shear rate are not correct: they are actually called “apparent shear rates”, and the resulting viscosity that is obtained by using them is called “apparent viscosity”. In order to retrieve the true viscosity, the apparent shear rates must be corrected using the Weissenberg-Rabinowitsch correction [38], a procedure that is described in many standard books [39]. Other important corrections are due to Bagley [40] and Mooney [41].

The Bagley procedure is necessary for taking into account entrance effects, i.e., the concentrated pressure losses sustained by the fluid in moving from the instrument reservoir to the capillary tube. Roughly speaking, it consists in running measurements with capillaries of different lengths at equal flow rate. This correction is normally mandatory but can be avoided in the case of slit viscometry, if pressure is measured through two or more transducers that are flush mounted along the slit [37]: in this case, the pressure measurement is not influenced by entrance effects, provided that the transducers are placed sufficiently far from the slit entrance. The measured pressure gradient can be directly input into the wall shear stress expressions of Table 1 as the ratio P/L.

The Mooney correction procedure is used for correcting wall slip effects that would underestimate viscosity. In fact, since the apparent shear rate as determined from the formulae in Table 1 is directly linked to the flow rate, it includes a wall slip contribution, which must be eliminated. The correction is performed using capillaries of different radii or slits of different depths at equal wall shear stress, and it provides other important information about the rheological behavior of the material, particularly the slip velocity as a function of the wall shear stress.

### 2.2. Rotational Rheometry

If the instrument is a parallel plate rheometer, the shear rate is not uniform. Its maximum value is for the radial coordinate equal to the plate radius R and can be directly related to the angular velocity of the moving plate Ω through the expression reported in Table 2, H being the gap between the two plates. If the fluid is Newtonian, the shear stress at the same location can be determined from the torque $M_{T}$ that is applied by the instrument and is also reported in Table 2. For the case of non-Newtonian fluids, it should be corrected with a Rabinowitsch-like procedure. The corresponding expressions for the case of the cone and plate geometry are also reported in Table 2, with α being the slope of the cone. This case is interesting because the shear rate is uniform in the whole fluid domain; thus, the shear stress is uniform, as well, and the correction for non-Newtonian effects is not necessary.

Rotational rheometers can be operated by imposing either a continuous rotating motion or an oscillatory one. If a constant angular velocity is superimposed, one can obtain useful information also about the normal stress differences of the tested fluid, particularly the first normal stress difference [39,42]. This can be done by measuring the normal forces that tend to separate the plates while running the test. The oscillatory operating mode makes it possible to obtain information about the viscoelastic properties that is interesting for extrapolating structural and morphological characteristics. After superimposing a sinusoidal shear strain of frequency ω/2π and amplitude $\gamma_{0}$

(2) $$\gamma =\gamma_{0}\sin\omega t,$$

it can be shown that the response of the material in terms of shear stress is also sinusoidal with the same frequency, but it is out of phase by a certain angle δ:

(3) $$\tau =\tau_{0}\sin\left(\omega t+\delta \right);$$

next, one can define the storage modulus $G^{\prime }$ and the loss modulus $G^{\prime \prime }$, as follows:

(4) $$G^{\prime }=\frac{\tau_{0}}{\gamma_{0}}\cos\delta \;\;\;\;\;\;\;\;G^{\prime \prime }=\frac{\tau_{0}}{\gamma_{0}}\sin\delta .$$

Taking into account that they are measured through oscillatory testing, they are also called dynamic moduli and are a function of frequency and temperature. In order for the above formulae to be significant, the fluid must be loaded within its linear viscoelasticity region (LVR) [39]. This requires that $\gamma_{0}\ll 1$, which is sometimes difficult to achieve. On the other hand, such a procedure does not require the corrections that are necessary when the rheometer is operated in continuous mode.

Viscoelastic characterization can be obtained from the evaluation of the dynamic moduli as a function of ω: the storage modulus is a measure of the elastic response of the fluid, while the loss modulus is related to energy dissipation. In particular, if the loss modulus is greater than the storage modulus, one talks about a viscoelastic fluid, while, if the converse is true, the material is considered a viscoelastic solid. The tangent of the phase angle is obtained by the ratio of the loss and storage moduli:

(5) $$\tan\delta =\frac{G^{\prime \prime }}{G^{\prime }},$$

and is called damping factor. A further quantity of interest is the complex viscosity:

(6) $$\eta^{*}=\frac{\sqrt{{\left(G^{\prime }\right)}^{2}+{\left(G^{\prime \prime }\right)}^{2}}}{\omega },$$

which is also a function of loading frequency and temperature. Despite having only the physical dimension of viscosity, it may be considered to be equal to the shear viscosity η through the so-called Cox-Merz rule [39,42]:

(7) $$\eta \left(\dot{\gamma },T\right)=\eta^{*}\left(\omega ,T\right),$$

with $\dot{\gamma }$ measured in s^−1^ and ω measured in rad/s.

### 2.3. Differences between the Two Techniques

The main difference between the two methods is the shear rate range that can be investigated: pressure-driven viscometers are used to explore the material behavior at high shear rates, while rotational rheometers deal with low to medium shear rate measurements. Despite the fact that the Cox-Merz rule could allow measurements at higher shear rates, its application may be questionable, and it also has some special requirements, in that the fluid must be loaded within its LVR. Moreover, rotational rheometers, unlike pressure-driven viscometers, are not suitable for characterizing fluids concerning processing defects and flow instabilities.

Nevertheless, rotational rheometers are more popular among researchers, possibly because their overall simplicity, since, when operated in oscillatory mode, the correcting procedures that are required by pressure-driven viscometry are not necessary. For instance, the application of the Mooney procedure, besides involving an increased complexity, may introduce significant measurement errors that could even compromise the accuracy of the rheological characterization [43,44].

## 3. Wood Polymer Composites Characterization with Pressure-Driven Viscometers

Pressure-driven viscometers can characterize fluids at high shear rates if narrow capillaries or slits are used. In the case of molten WPCs, though, the viscosity of these fluids may be very high, especially when the wood content is more than 50 wt.%; therefore, if the capillary is too narrow, pressure may become too high [13]. On the other hand, capillaries of different radii must be used for the Mooney correction, which is often necessary for molten WPCs: lubricants promote wall slip not only during processing but also during characterization; thus, their presence may alter viscosity measurements if wall slip is not taken into account properly. This situation is also worsened by pressure dependent slip phenomena occurring at the exit of pressure-driven viscometers [45,46].

An additional problem caused by the use of narrow capillaries is the sieving effect [47]. In some cases, this phenomenon can be very significant because the wood particles tend to aggregate in clusters that can be sieved and separated from the polymer matrix at the entrance of the capillary. If this occurs, the measured viscosity would be underestimated, or the pressure may display an oscillating trend due to fluttering phenomena. On the other hand, an important advantage of these viscometers is that the use of inert atmosphere during testing is not necessary. In fact, since these instruments work at a reduced oxygen content, the danger of WPC oxidative degradation is limited.

An interesting pressure-driven instrument is the in-line viscometer [37]: it is an ordinary single screw extruder mounting a slit die capable of evaluating the pressure drop by measuring pressure at specific locations. Its general advantages are that the material is characterized in real processing conditions, and, if a sufficiently long slit is used, the Bagley correction is not necessary. Moreover, these devices are particularly suited to characterize natural fiber filled polymers, since they handle the material at a very low oxygen quantity and they do not have any filler sieving problems. Large shear rates are normally not achievable, but they are obviously in the same range as those of extrusion.

In literature, many authors have characterized WPC with pressure-driven viscometers. The formulations are listed in Table 3 and the testing parameters are reported in Table 4. In particular, the wood flour concentration ranged between 5 wt.% and 70 wt.%, and the most widely used additives are coupling agents and lubricants. Testing temperatures ranged between 160 °C and 240 °C, but the most widely used one is 180 °C. In both tables, the bibliographic reference is reported in the last column.

All papers agree about the non-Newtonian nature of these fluids, with a shear thinning behavior that is present in all systems [13,43,48,49,50,51,52,53,54,55]: viscosity depends on the shear rate and decreases with it. This is typical of high molecular weight fluids, where it is due to the secondary bonds (entanglements) among macromolecules being continuously broken and reformed during the flow. At elevated shear rates, these bonds do not have enough time to rebuild, leading to a viscosity decrease. In the case of WPCs, this is further complicated by the wood fibers playing a role in the viscosity modification as the shear rate increases, for example, due to fiber orientation phenomena [56]. Slip velocity can also be characterized through pressure-driven viscometers [31,33,35,36,37,43,46,52,54,57,58,59,60,61], and it is well established that it is an increasing function of the wall shear stress.

### 3.1. Wood Flour Effects

Wood flour can considerably influence the rheology of WPCs. The natural filler related parameters that have been carefully studied in the literature are the amount, type, and morphology of the fibers and their pretreatments. The interaction with the matrix is often described in correlation with these factors.

Many papers investigated the effect of wood flour concentration on the shear or apparent viscosity [33,43,49,50,52,54,56,57,59,60,62,63,64,65,66]. Everyone agrees that viscosity increases with concentration, regardless of all other factors. Viscosity can be as high as 1–10 kPa s in the case of 50 wt.% wood loading at low shear rates (100 s^−1^). The wood content has also a great influence on the wall slip behavior, with slip velocity increasing with the amount of natural filler [33,43,52,54,58,59,60,61,67]. Slip velocity is of the order of 1–10 mm/s at a 200 kPa applied shear stress.

The wood type, i.e., whether from conifers or from deciduous trees, may also have an important influence. Li and Wolcott [43] studied the dependence of the rheological properties on the wood species by using two different HDPE-based WPC systems: one from maple (a deciduous tree), the other from pine (a conifer). Interestingly, the two composites had different levels of wall slip, the pine-based WPC showing a higher slip velocity. The authors explained this with the presence of a significant content of fatty acid in pine wood that could have migrated to the surface and acted as an external lubricant. The wood species can also influence the shear thinning behavior, with maple showing a more pronounced effect than pine.

Many studies have dealt with the effect of particle size [50,51,53,57,59], indicating that as the dimensions of the wood particles decrease, the apparent viscosity increases [53,57,59,61]. This can be justified because at equal shape fine particles expose more surface area than coarse particles, and this favors the probability that more chemical bonds be created with the matrix. This is valid if the wood content is lower than 45 wt.%, while, at higher wood loading, viscosity increases irrespective of the decrease in the particle size [57,59]. Hristov [61] evaluated the influence of particle dimensions on wall slip, observing that small particles imply a much lower wall slip than coarse particles, and this behavior seems to be due to differences in the wall slip layer thickness: larger particles induce a thicker layer, which leads to a higher apparent slip velocity.

The wood particles chemical treatments are also of interest [68,69,70,71,72]. These are primarily used to increase the mechanical properties of the material, enhancing the interfacial adhesion between the reinforcement and the polymeric matrix. The most common chemical modifications of wood particles are alkalization [71], silane treatment [72], and esterification [69]. Alkaline treatment eliminates or reduces the amount of lignin and hemicellulose in wood flour, thereby improving the surface quality of the product. Nonetheless, the refinement of natural fiber diameter was also considered to explain the reinforcing effects [17]. Alkaline treatment results also in an increase in the shear viscosity [71], and similar results were obtained with a potassium permanganate (KMnO_4_) treatment that increases the surface roughness and, therefore, the interfacial adhesion between the wood particles and the matrix [68]. Other treatments are less univocal, as the effects on viscosity depend on the shear rate. One example is with an ionic liquid treatment, used as a solvent, that increases the viscosity at low shear rate but acts as a lubricant and a plasticizer at higher shear rates, thereby reducing viscosity [70]. The opposite happens with a citric acid treatment that causes a reduction in viscosity compared to the untreated fiber filled WPC at low shear rates, while, at high shear rates, the decrease is less significant, because the particle-particle interaction decreases [69].

Interestingly, also mechanical treatments on the wood particles were considered. Unfortunately, though, only one paper dealt with this subject: Li et al. [62] observed a significant decrease in the viscosity when performing a grinding treatment at 80 °C for 2 h before extrusion. Despite the fact that these results seem to be in disagreement with fiber refinement obtained through chemical treatments or usage of finer particles, they should be actually interpreted as coming from a thermo-mechanical treatment.

### 3.2. Polymer Matrix and Temperature Effects

As can be seen from Table 3, most of the studies on the rheological properties of WPCs used HDPE as matrix. Since this polymer melts at a relatively low temperature (about 135 °C), HDPE-based WPCs are probably the most easily processable materials. On the other hand, numerous papers dealt with PP-based WPCs: this material has a lower density and the important advantage that the mechanical properties are generally better than HDPE. On the back side, the melting temperature is about 165 °C; thus, the processing window is rather narrow. Concerning the rheological properties, though, not many differences can be noticed between HDPE-based and PP-based WPCs. Perhaps, the polymer molecular characteristics are more important in determining the rheology of the composite.

Only a few papers compared how the molecular weight of the polymer matrix and its distribution can influence the flow characteristics of WPCs [59,61]. Hristov and Vlachopoulos [59] observed that the shear viscosity increases using a lower molecular weight matrix. This unexpected result was explained with a better wettability of the wood particles that promotes an improvement in the mechanical adhesion, leading to a resistance to shearing. Moreover, the molecular weight influences the wall slip behavior: in fact, comparing two metallocene HDPEs with the same wood content (i.e., 30 wt.%), polydispersity index, and particle dimension, the polymer with a lower molecular weight seems to have a higher wall slip, and the authors justified this result with a lower viscosity of the apparent slip layer [61].

Another parameter that needs to be taken into account is the effect of the testing temperature. Although the reinforcement is solid, the viscosity of molten WPCs reduces significantly at higher temperatures because the movement of the polymeric chains are enhanced [55]. On the other hand, this behavior is predictably less evident as the percentage of wood increases [50]. The equation that is often used to model the relationship between viscosity and temperature is the Arrhenius equation:

(8) $$\ln\eta_{0}=\ln K+\frac{E_{\eta }}{RT},$$

where$\;\eta_{0}\left(T\right)$ is the zero shear viscosity, K is a material constant, R is the gas coefficient, $E_{\eta }$ is the activation energy, and T is the absolute temperature [55].

### 3.3. Influence of the Additives

Several studies focused on the choice of the most suitable additives to obtain a product with optimal physical and mechanical performance. The most important additives of WPCs are coupling agents and lubricants [48,53,58,61,63,64,67,68,73].

The best coupling agents in the case of polyolefins are low molecular weight variants of the polymer that is used as the matrix, modified with maleic anhydride (MAH). Examples are MAH-modified polyethylene (MAPE) or polypropylene (MAPP). Often these additives are compounded in small amounts (1–5 wt.%) and have the side effect of functioning as an internal lubricant [58], but this is still a source of debate: in some papers, accordingly, there is a viscosity decrease [58,64,73], while, in others, the opposite trend is observed [60]. In the first case, the justification stems from the plasticizing effect of the coupling agent, while, in the second case, the explanation derives from the improvement in the fiber-matrix adhesiveness, which would produce a greater resistance to shearing.

Lubricants are processing aids that improve the surface quality of the product, decrease wood degradation and limit energy consumption during the forming phase. Common lubricants are fatty acids, esters, stearates, and silicones. Unlike internal lubricants, that decrease viscosity by acting as plasticizers [63], these compounds have a reduced solubility; thus, they place themselves between the fluid and the die wall, favoring wall slip, and are therefore more properly indicated as external lubricants. Generally, the studies that concern this topic are based on the choice of a specific type of lubricant and focus on finding the most appropriate percentage [48,63], by comparing lubricated systems with non-lubricated ones [53,67] or evaluating different lubricating systems [58]. An adequate lubricant reduces the apparent viscosity [53,58,61,67], and this effect increases with its content. This in general ranges 1–10%, depending on the type of lubricant [48,63]. Hristov and Vlachopoulos [67] also performed the Mooney procedure for the determination of slip and found that the addition of a 1 wt.% of lubricant leads to a significant increase in the slip velocity. This effect is more significant at low wall shear stresses and is very convenient in the case of WPC, which is usually extruded at low velocities and shear stresses. In addition,, Li and Wolcott [58] compared two different lubricating systems, i.e., an ester-type and zinc stearate, and observed that the reduction in viscosity with the ester-type lubricant is much more important than the stearate-based system, confirming that zinc stearate is not compatible with coupling agents based on MAH [74].

### 3.4. Modeling, Processing and Surface Defects

Several authors [43,48,49,50,52,53,55] modeled the shear viscosity of WPCs with a suitable shear thinning constitutive law. The model that is most widely used is the Ostwald-DeWaele power law [75]:

(9) $$\tau =k{\dot{\gamma }}^{n},$$

where k is the consistency index, and n is the power law exponent. Consistency can have a very wide range (e.g., 10–1000 kPa s), depending on composition and testing temperature [43,52,55], while the power law exponent is relatively low (e.g., 0.3–0.45) and decreases with the wood content [43,49,52,53,56] and with the dimension of the wood particles [50]. Since a lower power law exponent is linked with a higher shear thinning behavior, higher wood flour fillings correspond to more shear thinning materials.

An interesting work is the one by Santi et al. [53], in which the results obtained with a capillary viscometer were compared with a slit viscometer. Both curves were fitted with a power law equation and the results show a similar power law exponent, while consistency has a higher variability. These differences became very significant when a lubricant was added into the compound.

The shape of the viscosity curve may change depending on the wood content. At low wood flour percentages, a Newtonian plateau is visible at low shear rates [43]; therefore, the Carreau-Yasuda equation is more adequate to fit the viscosity data [76]:

(10) $$\frac{\eta \left(\dot{\gamma }\right)-\eta_{\infty }}{\eta_{0}-\eta_{\infty }}={\left[1+{\left(\lambda \dot{\gamma }\right)}^{a}\right]}^{\left(n-1\right)/a}.$$

In addition to the shear thinning exponent, in this relationship, there are 4 more additional parameters, namely the zero-shear viscosity $\eta_{0}$, a time constant λ, a fitting parameter a and the infinite shear viscosity $\eta_{\infty }$, which is normally taken to be equal to 0. Li and Wolcott [43] further observed that, at a 60 wt.% wood content, a small upward pointing cusp appears at very low shear rates, which led them to suppose the presence of a yield stress. On the other hand, as the typical shear rate to appreciate the presence of the yield stress is rather low, the rotational rheometer is perhaps a better instrument to infer the presence of this phenomenon. More on this issue will be presented in the next section.

One of the main interests regarding the study of rheology is its impact on WPC processing, in particular concerning extrusion. Extrusion is arguably the most important processing technique, because the main application of WPC is the realization of profiles that would substitute natural wood boards. This is in line with the advantage of filling WPC with high amounts of wood flour, which would not allow processing by injection molding. Nevertheless, these materials are not easy to process, even by extrusion, as they are not homogeneous and often show flow instabilities, surface tearing and severe melt fracture [52,59,61,67,70,71]. Hristov and Vlachopoulos [61,67] attributed these defects to a large difference in extensional rate between the core and the surface of the extrudate. These effects can be limited by enhancing wall slip, that can be achieved through either increasing the apparent shear rate, or adding external lubricants, or using a wood content greater than 50 wt.%.

A good and easy solution to increase the surface quality and the throughput is to increase the apparent shear rate (i.e., the expressions in Table 1) because the material tends to a plug flow regime. In fact, WPCs show three flow regimes: at low shear rates (below 100 s^−1^) where the flow is characterized by a weak slip and extrusion defects appear, at medium shear rates (100–500 s^−1^) with intermediate characteristics, and, at high shear rates (above 500 s^−1^), where the material is in complete plug flow conditions and the extrudate surface morphology becomes of good quality [52,59,61,70]. Ou et al. [71] named these three regimes “sharkskin”, “stick-slip”, and “super extrusion”, respectively.

On the other hand, increasing the shear rate is not always possible: extrusion is not a fast process, and, in fact, the typical maximum shear rates that can be reached are slightly under 100 s^−1^. The best solution to limit melt defects, thus, is to introduce an adequate content of external lubricants. This is very common and facilitates the extrusion process remarkably [61,67]. The effect of filler concentration and size was studied in [52,54,61], confirming that at equal shear rate, higher wood percentages (i.e., more than 50 wt.%) make surface defects almost disappear. This effect is especially favored when using coarse wood particles [61]. Furthermore, it should be added that the extrusion defects are strongly influenced by the design of the die. In particular, surface tearing effects are more evident at higher die sizes [54,65], possibly because of a lower forming pressure within the die.

## 4. WPC Characterization with Rotational Rheometers

Despite the fact that there are many papers in the literature that use rotational rheometers for the rheological characterization of molten WPCs, this technique has a few drawbacks that are specific of natural fiber filled polymers. First of all, testing should be performed in inert atmosphere to prevent oxidative degradation of lignin and hemicellulose. Next, the cone and plate geometry cannot be used due to the high viscosity of the fluid, and this also prevents testing in continuous mode, unless the material contains very low filler amounts. On the other hand, if testing in oscillatory mode is employed, the material must be loaded within its LVR, which for molten WPCs is well known to be very narrow and to occur at very low strains [13,77,78,79,80]. Moreover, the validity of the Cox-Merz rule is anyway questionable, as it should not be applied to concentrated polymeric suspensions [81]. The advantages of this technique, though, are very convenient: it is simple, does not require cumbersome corrections, there are no fiber sievage problems, and wall slip has a very limited influence on the rheological measurement in oscillatory mode. Perhaps, though, the main benefit is the possibility of obtaining additional structural information concerning interface properties and fiber dispersion quality.

The compositions of the materials and the testing parameters that have been studied so far, are reported in Table 5; Table 6, respectively. The same general remarks that were put forward for pressure-driven viscometers apply also to the case of rotational rheometers: the wood flour content ranged between 10 wt.% and 70 wt.% and again the most widely used additives are coupling agents and lubricants. Testing temperatures varied between 140 °C and 210 °C, the most common one being 180 °C.

**Figure 1:**
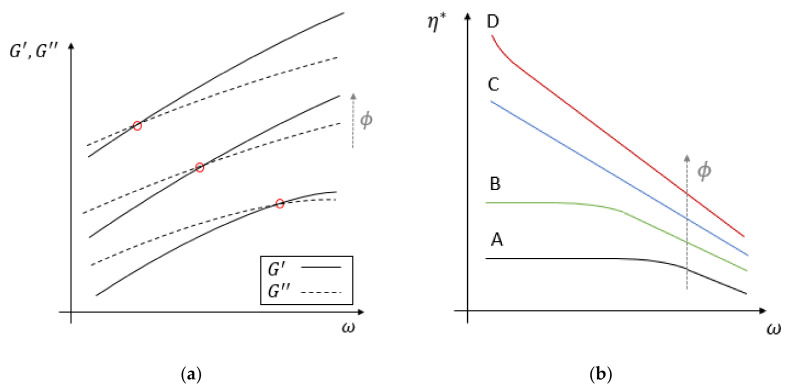
(**a**) Typical dynamic moduli plots ($G^{\prime }$ solid line, $G^{\prime \prime }$ dashed line) for increasing wood flour content ϕ. The cross-over points (red dots) shift at lower frequencies as the wood content ϕ increases; (**b**) Typical complex viscosity plot at increasing wood flour content, showing the Newtonian-like plateau disappearing as the wood content ϕ increase (curves A, B, and C). Yield may appear at very high wood flour content (curve D).

In the vast majority of the papers, the rheometer is operated in oscillatory mode; hence, the main results that are reported are the plots of the dynamic moduli and the complex viscosity as a function of frequency. Often, at very low frequencies, the loss modulus is greater than the storage modulus, i.e., the material behaves like a viscoelastic liquid. On the other hand, at the typical testing temperatures, both dynamic moduli increase with frequency, and, since the storage modulus increases faster than the loss modulus, at a certain frequency a cross-over is reached (Figure 1a). This means that the material changes its behavior, i.e., it switches from that of a viscoelastic fluid at low frequencies to that of a viscoelastic solid at higher frequencies [14,18,55,72,77,78,79,82,83,84,85,86,87,88,89]. The complex viscosity has a decreasing trend with frequency, similar to a shear thinning fluid, if the Cox-Merz rule was supposed to hold (Figure 1b, curve A). This behavior is indeed common to the unfilled matrix, in which it is the result of the formation and disruption of a relatively small number of secondary bonds among the polymeric chains (e.g., chain entanglements) [54,80,90]. Nevertheless, the presence of the natural filler is responsible for a number of additional phenomena that make the interpretation of experimental data more complex. These can be understood by comparing the rheological behavior of molten WPCs with that of the unfilled matrix, as performed in almost all papers.

Wood particles may give rise to two types of interactions: filler-filler and filler-polymer. The former ones are responsible for wood particle clustering or agglomeration and may have a strong influence on the final properties since they contribute to creating a non-soluble structure within the material. The net result is that flow is possible only after such a structure is destroyed by a sufficiently high applied stress, a phenomenon commonly known as yield [91]. The latter ones are responsible for an increasing difficulty for polymer chains in disentangling from one another. This has two consequences: on one hand, the material has more internal bonds; on the other one, once a secondary bond is broken, it is more difficult to reform it. The net result is a higher shear and complex viscosity, as well as higher dynamic moduli, but also a Newtonian-like plateau in the complex viscosity curve that terminates at a lower frequency (Figure 1b, curve B). The subsequent decay with frequency (similarly to the shear thinning behavior) is steeper in the case of wood filled polymers, and this can be explained by the reduced room available for the polymer chains, due to the presence of the wood fibers [55,92]. The behavior becomes anyway more solid-like at higher frequencies because the polymer chains have less time to disentangle [52].

### 4.1. Wood Flour Effects

Like in the case of pressure-driven viscometers, the wood flour content has a great influence on the rheological behavior of WPCs. The storage modulus increases with the wood percentage and so does the loss modulus and the complex viscosity [13,55,65,72,83,86,87,93,94]. This behavior is primarily a consequence of the filler-filler and filler-polymer interactions as described previously.

At low frequencies (i.e., less than 1 rad/s), if the wood content is below 30 wt.%, the complex viscosity curve displays a Newtonian-like plateau [14,92], and the behavior is mostly influenced by the polymer-polymer interactions. At higher wood content, filler-polymer interactions become more important; thus, the Newtonian plateau reduces and tends to disappear [13,65,77,89,92,95], as shown in Figure 1b (curve C). At even higher wood percentages, filler-filler interactions are dominant, and yield phenomena may appear in some systems (Figure 1b, curve D). In confirming this, Yang et al. [92] found that there exists a critical wood content where a network is formed inside the material: below this value, the WPC behavior is more similar to that of a dilute suspension, while, above this value, a 3D-structure made by the wood particles is stable.

At high frequencies, the behavior is qualitatively similar for all wood percentages, with the complex viscosity showing a shear thinning like behavior, that can be modeled as a power law. The effect of the wood particles is limited to the curve becoming steeper at higher filler content (Figure 1b) [14,15,53,54,72,77,79,85,86,90,92,93,94,96]. The rheological behavior is, thus, anyway more dependent on the characteristics of the polymeric matrix.

Concerning the dynamic moduli, at increasing wood content, the material has a more solid-like behavior, in that the $G^{\prime }$ and $G^{\prime \prime }$ crossover frequency shifts at lower frequencies (Figure 1a) [13,54,90,92]. Indeed, at sufficiently high wood content and loading frequency, the storage modulus is always greater than the loss modulus and depends on the particular formulation of the material. Recalling Equation (5), the damping factor decreases with the filler increment indicating that the viscous dissipation of WPC is greater than that of the neat matrix. Moreover, when the percentage of wood particles is limited, this parameter decreases linearly and with a high slope. When the filler content increases above 30 wt.%, a peak in the damping factor plot may appear [79,92].

Particle size is another important factor that was considered. The complex viscosity and the dynamic moduli seem to be slightly influenced by the dimension of the wood particles when the size variation is moderate [77] and the frequencies are high [59], while the complex viscosity increases at low frequencies when the filler morphology is constituted by small particles [53,59]. Moreover, this trend becomes more important at high percentages of wood. This can be explained because, as discussed previously, at low frequencies the rheological properties are dominated by the behavior of the filler, while, at high frequencies, they depend more on the matrix.

Some researchers have evaluated the effect of pretreatments on the wood fibers before the compounding phase. Typically, the treatments can be either chemical [69,77] or thermal [55,83], but both aim at modifying the fiber surface and improving the bonding mechanism between matrix and reinforcement. A widely used chemical treatment is the alkaline treatment [17,19,71,77], which reduces the content of lignin and hemicellulose, making the surface of the fiber rougher and increasing the number of reactive hydroxyl groups [71]. In terms of rheological properties, this treatment slightly decreases the complex viscosity and the storage modulus [77]. The treatment based on citric acid also causes a reduction in complex viscosity and dynamic moduli, especially at low frequencies (0.1–1 rad/s). Moreover, the slope of $G^{\prime }$ and $G^{\prime \prime }$ curves increases, denoting a better dispersion of the filler [69]. On the other hand, there are pretreatments that can increase both $G^{\prime }$ and $G^{\prime \prime }$ and complex viscosity, like, for example, using an ionic liquid. Furthermore, this treatment tends to stabilize a sort of yield stress even at relatively low wood flour content, as it is evident from the presence of a small plateau in the dynamic moduli curves at low frequencies [70].

Thermal treatments consist in a heating phase that can take place either in boiling water or in inert atmosphere (e.g., nitrogen or argon) and often work by cracking or modifying lignin and hemicellulose. The generalization of these results is not easy because they can influence the complex viscosity in different ways: complex viscosity can increase with thermal treatments [83,97] but may also decrease [55].

### 4.2. Polymer Matrix and Temperature Effects

HDPE and PP are the most commonly investigated matrices with rotational rheometers (Table 5), but, in the last few years, some biodegradable or compostable matrices were also considered, such as PLA [14,18,88] or vegetable oil-derived polymers, like Mater-Bi [15]. The results in terms of rheological characterization are similar to those of more common materials. The only differences are that biodegradable matrices usually accept only limited amounts of wood flour, i.e., 30–40 wt.% maximum, possibly because of the higher chemical affinity between these polymers and natural fibers [18,88]. It can then be speculated that even a 10 wt.% is a relatively large wood content for these materials. Nevertheless, the effect of natural filler content on the complex viscosity seems to be comparatively stronger, and, in particular, with Mater-Bi, the appearance of a yield stress at low frequencies, even at a mere 15 wt.% wood loading, seems incontrovertible [15]. Moreover, one should not forget that these polymers must be dried carefully before compounding with natural fibers and testing [16].

Some papers evaluated the effect of matrix recycling on WPCs based on PP [90] and on HDPE/PP blends [98] using rheological characterization. These studies are very important to evaluate matrix degradation phenomena and their interaction with the wood fibers after the compounding phase. Although there are no large differences between the storage modulus curves of the virgin polymer and the samples containing 50 wt.% of unfilled recycled polymer, the addition of the natural filler leads to a decrease in both the storage and loss moduli when a certain amount of recycled polymer is compounded in the formulation. Twite-Kabamba et al. [99] evaluated the effect of WPC recycling in a closed-loop reprocessing procedure up to ten times, comparing the results with the neat matrix that followed the same number of cycles. WPCs complex viscosity decreases by about 30% with ten reprocessing cycles, indicating that molecular weight has been reduced.

Temperature can also influence the dynamic properties of WPCs: it was observed that, at low temperatures, the dynamic moduli increased following a similar trend as the neat matrix [86,93]. Some authors justified this behavior because wood is hydrophilic, and the presence of internal humidity can make the complex viscosity more sensitive to temperature compared to the neat matrix [93]. Other authors observed this trend to be less significant, especially at higher wood content, explaining this with the formation of particle aggregates that limit the movement of macromolecules [92].

Temperature effects were taken into account in [18] and [88], using the Williams Landel Ferry equation [100]:

(11) $$\log\left(a_{T}\right)=-\frac{C_{1}\left(T-T_{0}\right)}{C_{2}+\left(T-T_{0}\right)}\;,$$

in which $a_{T}$ is the thermal shift factor, $C_{1}$ and $C_{2}$ are fitting parameters, and $T_{0}$ is the reference temperature.

### 4.3. Influence of Additives

Like in pressure-driven viscometry, coupling agents [77,79,82,90,101,102,103] and lubricants [58,95,101] are the additives that were mostly investigated with rotational rheometry. Usually, the main coupling agents that are used are MAPE and MAPP when HDPE or PP are used as matrix [60,77,79,82,101,102,103] but their effect on the rheological properties is still debated. Bi et al. [82] tested numerous additives on a TPU-based WPC, finding that the MAH-modified TPU seems to increase the storage and loss moduli and the complex viscosity. Similar results were obtained using MAH in different polymeric systems [77,79,98,101,102] and were interpreted as an improvement in the adhesion between wood and matrix. A different interpretation was made by Li and Wolcott [58], who associated the best performance to a system based on an ester-type lubricant and MAH, in which the decrease in complex viscosity and $G^{\prime }$ is significant. A similar observation was made by Gao et al. [103], who compared a compatibilized WPC with a material formulation without coupling agents. In addition, they evaluated the MAH content and observed that, when the additive percentage reaches a certain value, the dynamic moduli and the complex viscosity start again to increase.

Another typical WPC coupling agent is silane [72,90,96], and its effect is strictly dependent on the wood content: no effect is appreciated in the storage modulus for a 10 wt.% wood loading and with 0.5 and 1 wt.% of silane [90]. As the wood content increases (20–30 wt.% or more), the particles have more probability to come in contact with each other, but, if the silane is present, the number of agglomerates tends to decrease, favoring flow and chain movement, thus reducing the storage modulus. By increasing the quantity of additive, the storage modulus slightly increases, and this can be explained by an improvement of the filler-matrix interface [72]. An alternative to improve the compatibility between the polymer matrix and the wood fibers is the addition of graft copolymers [55,104]. As for MAH, the results are not always in agreement: sometimes they increase complex viscosity [55], and, in other systems, the behavior is the opposite [104], but, in general, the effectiveness in improving the interfacial interactions between polymer and reinforcement is confirmed.

Lubricants are also very common and their effects are studied also with rotational rheometers [58,95,101]. Stearic acid, semi-refined paraffin wax, and zinc stearate reduce complex viscosity, and this effects increases with the increase of lubricant content, but the best compromise seems to be obtained by combining stearic acid with wax lubricants [95] or with MAH coupled with ester-type lubricants [58,101]. In the first system, stearic acid decreases the polarity of the wood particles, while wax acts as an internal lubricant in the second system. An important conclusion is that ester-type lubricants are an excellent external lubricant that does not neutralize MAH as a coupling agent. This instead happens with zinc stearate that reacts with MAH chemically and increases the complex viscosity dramatically, deactivating their respective functions [58].

Habibi et al. [98] evaluated the effect of EPDM as a toughening agent to improve the impact resistance. Surprisingly, the addition of the toughening agent produces a storage modulus increase, especially at low frequencies. This effect seems to be due to a better interaction between the different phases.

### 4.4. Modeling, Processing and Structural Properties

A power law equation (11) is often applied to facilitate the interpretation of the experimental data in terms of complex viscosity [54,90,92]. The power law exponent decreases with filler content, confirming the shear thinning-like behavior also in the case of dynamic viscosity measurements [90,92]. The most widely used justification for this behavior is a reduction in the polymer layer between the wood particles, making WPC more sensitive to shear deformations [54,92]. Accordingly, the complex viscosity rises considerably with a wood content increase. As for the data obtained with pressure-driven viscometers, the presence of a yield stress is controversial [54,91]. Complex viscosity can also be interpreted with a Carreau-Yasuda equation (12) if a plateau at low frequencies is visible, obtaining a zero shear viscosity $\eta_{0}$ that is strongly dependent on the wood content [13,35,83] or on the number or reprocessing cycles sustained by the WPCs [99].

In some papers, a single master curve for the complex viscosity vs. frequency was obtained. This idea, initially proposed by Highgate and Whorlow [105] and described also by Barnes [106], allows to predict the flow curves for filled viscoelastic system at any composition and temperature. In case of WPCs, this possibility is very intriguing because natural fiber filled polymers are materials that are very difficult to characterize. The procedure can be thought of as an extension of the Williams Landel Ferry theory [100], to include filler amount in addition to temperature. If the master curve is indicated with $\eta^{*}{}_{\mathrm{ref}}$, the complex viscosity values at any frequency ω, wood content ϕ, and temperature T can be obtained by using appropriate shift factors, such as a thermal shift factor $a_{T}$ and a concentration shift factor $b_{\phi }$ [13,49,80]. One expression that was proposed is [18]:

(12) $$\eta^{*}\left(T,\phi ,\omega \right)=a_{T}b_{\phi }\eta^{*}{}_{\mathrm{ref}}\left(a_{T}b_{\phi }\omega \right).$$

Interesting information regarding processing can also be obtained through rotational rheometry. Askanian et al. [97] measured the complex viscosity and the dynamic moduli of two different WPCs possessing the same wood content but that were obtained by extrusion at two different screw speeds (i.e., 500 and 1200 rpm), resulting in no significant difference. It can be concluded that this processing parameter has no influence on the dynamic properties, at least at relatively high screw speeds.

Zhang et al. [107] investigated the effects of extruder screw profiles on the preparation of 50 wt.% WPC compounds. They used three different screw profiles in a twin-screw extruder, changing the number of kneading or turbine elements in order to favor a distributive or a dispersive mixing or an average between the two mixing types. They measured the repeatability of seven complex viscosity measurements with all screw configurations and considered the effect of the screw speed (50 up to 200 rpm) and the presence of a lubricant. They concluded that the best screw choice is the one that provides an intermediate value of dispersive and distributive mixing. The lubricant is fundamental for this material from the processing point of view and the screw speed strongly influences the miscibility, fixing at 100 rpm the best material uniformity.

## 5. Discussion and Conclusions

Despite the fact that WPCs are relatively novel materials, there is a significant amount of literature concerning their rheological properties. The most widely investigated materials are the polyolefin-based ones, in which general properties are better understood, in terms of their basic rheology and interactions with filler and additives. Rheological studies about completely bio-based materials, such as PLA-based WPCs, are still in their infancy, but it can be easily foreseen that these materials will soon attract the attention of both research and consumer market.

Concerning rheological properties, an interesting conclusion that can be drawn from the scientific literature that made use of rotational rheometers is that at low frequencies the rheological properties are dominated by the behavior of the filler, while, at higher frequencies, they depend more on the matrix. Rotational rheometry studies are also extremely important for evaluating the quality of compounding. In fact, these instruments can measure viscoelastic properties that are heavily dependent on structural characteristics, such as filler-matrix interactions, wood flour dispersion, and additives influence and their side effects. These, in turn, depend on material formulation (e.g., additives, wood amount, polymer molecular weight), and compounding variables (e.g., twin screw profile, temperature, screw speed) and their complex interplay. As a consequence, these studies are an invaluable help for understanding how a material with the best performance can be obtained as a compound. On the other hand, it is hard to generalize these results, as they are very specific for the particular system that is being investigated; therefore, what is described in the literature should be interpreted more as a methodology to follow in pursuit of optimization, rather than a detailed guide for obtaining the best recipe.

The general results that have been found are displayed schematically in Table 7. Wood content is the variable that mostly influences rheological properties, which increase remarkably with it. Anyway, both shear viscosity and complex viscosity are relatively high and decrease with shear rate and frequency, respectively. More valuable information about processing, though, can be obtained from the measurement of slip velocity, which can only be performed with a pressure-driven viscometer. Wall slip, actually, may well be interpreted as being more important than viscosity because it is probably the single factor that mostly influences surface quality and possible defects of the final product: a flawless product with a smooth surface can be obtained by clinching the optimal combination of wood percentage, additives, temperature, screw speed, and flow rate that enhances a strong slip or plug flow behavior. In this respect, it is well accepted that the slip velocity increases with shear stress, wood content, and the addition of an external lubricant.

On the other hand, we feel that more studies should be directed towards explaining the role played by the additives, in particular coupling agents and lubricants (see Table 7). This would be important to understand the link between processing variables, formulation and rheology, which can be extremely useful for obtaining a good quality final product. In fact, a last issue to address is the actual generalizability of the rheological characterization results. Typically, rheological measurements are performed on fluid specimens that are tested in a laboratory setting, often quite different from “real life” processing conditions. For such a reason, in-line rheological measurements, i.e., rheological testing procedures that are performed within a manufacturing process, are of particular interest for the designer of the manufacturing equipment and should be preferentially investigated in the next-to-come scientific papers.

## Figures and Tables

**Table 1:** Pressure-driven viscometers formulae. Q is the volumetric flow rate, *P* is the pressure drop, L is the capillary or slit length, R is the capillary radius, W is the slit width, and H is the slit depth.

Rheometer Geometry	$\mathrm{Wall}\;\mathrm{Shear}\;\mathrm{Rate}\;{\dot{\gamma }}_{w}$	$\mathrm{Wall}\;\mathrm{Shear}\;\mathrm{Stress}\;\tau_{w}$
Capillary	${\dot{\gamma }}_{w}=\frac{4Q}{\pi R^{3}}$	$\tau_{w}=\frac{PR}{2L}$
Slit	${\dot{\gamma }}_{w}=\frac{6Q}{WH^{2}}$	$\tau_{w}=\frac{PH}{2L}$

**Table 2:** Rotational rheometry formulae. R is the plate radius, Ω is the angular velocity, $M_{T}$ is the torque, α is slope of the cone, and H is the parallel plate gap.

Rheometer Geometry	$\mathrm{Shear}\;\mathrm{Rate}\;\dot{\gamma }$	Shear Stress τ
Parallel plate	${\dot{\gamma }}_{R}=\Omega \frac{R}{H}$	$\tau_{R}=\frac{2M_{T}}{\pi R^{3}}$
Cone and plate	$\dot{\gamma }=\frac{\Omega }{\alpha }$	$\tau =\frac{3M_{T}}{2\pi R^{3}}$

**Table 3:** Wood polymer composites (WPC) formulations characterized with pressure-driven viscometry in the literature.

Matrix	Wood Type	Wood wt.%	Coupling Agent	Lubricant	Other Additives	Ref.
**HDPE ^1^**	Poplar	40	/	/	/	[69]
	Poplar	37, 42, 47 52, 57	3% MAPE ^7^	/	5–20% CN ^8^	[62]
	Poplar	40	/	/	/	[70]
	Poplar	65	/	/	/	[71]
	Beech	20, 30, 40, 50	5% MAPE	/	/	[52]
	Pine	20, 30, 40, 50, 60	3% MAPE	2–10% TPN ^9^-709	/	[63]
	Eucalyptus	50	7% MAPE	/	/	[55]
	Pine	35, 40	4% MAPE	5% ester-type	/	[53]
	Maple	30, 40, 50, 60, 70	3% MAPE	/	/	[57]
	Maple	55, 57, 58, 60	2% MAPE	2% Zn stearate 1% EBSA ^10^ 2.7% ester type	/	[58]
	Maple-Pine	40, 60	/	/	/	[43]
	Maple	30, 45, 60	/	/	/	[59]
	Maple	30, 45, 60	1% MAPE	/	/	[60]
	Maple	5, 10, 20, 30, 50, 60	/	/	/	[65]
	Maple	25, 50, 60, 70	/	/	/	[54]
	Maple	30, 45, 60	/	TPS ^11^	/	[61]
**LDPE ^2^**	Lignocel	10, 70	/	/	/	[49]
**PP ^3^**	Birch	40	/	1.8–3% Struktol	0.33% Aox ^12^ and TS ^13^, 0.2–1% UVS ^14^	[48]
	Lignocel	10, 70	/	/	/	[49]
	White fir	30	/	/	/	[13]
	White fir	50	/	/	/	[46]
	White fir	35	/	/	50% TPE ^15^	[31]
	White fir	50–70	/	/	/	[35]
	White fir	30	/	/	/	[37]
	White fir	10, 70	/	/	/	[36]
	Pine-Firewood	50, 70	/	/	/	[33]
	Wood flour	25	1% MAPP ^16^	/	/	[73]
	Wood flour	30, 60	3% MAPP	/	/	[64]
	Wood flour	50	3% MAPP	1% TPS	/	[67]
	Wood flour	30, 40, 50	/	/	/	[56]
	Wood flour	9, 16, 23	/	/	/	[66]
	Maple	30, 45, 60	/	TPS	/	[61]
**ASA ^4^**	Bagasse	50	/	/	0–15% SBR ^17^	[68]
**PS ^5^**	Poplar	10,20,30,40,50	/	/	/	[50]
**PVA ^6^**	Whitewood	30	/	/	/	[51]

^1^ High density polyethylene, ^2^ low density polyethylene, ^3^ polypropylene, ^4^ acrylonitrile-styrene acrylate, ^5^ polystyrene, ^6^ polyvinyl alcohol, ^7^ modified polyethylene, ^8^ cellulose nanoparticles, ^9^ commercial lubricant, ^10^ ethylene bis(stearamide), ^11^ thermoplastic silicone, ^12^ antioxidants, ^13^ thermal stabilizer, ^14^ ultraviolet stabilizer, ^15^ thermoplastic elastomers, ^16^ modified polypropylene, ^17^ styrene butadiene rubber.

**Table 4:** Test conditions imposed with pressure-driven viscometry in literature. “S” stands for slit rheometer, and “B”, “M”, and “R” stand for the Bagley, Mooney, and Rabinowitsch correction procedures, respectively.

Temperature (°C)	Shear Rate (s^−1^)	Capillary/Slit Geometry (mm)	Corrections	Ref.
170	20–2000	1, 2	B	[69]
/	50–350	/	/	[62]
175	20–2000	1	B	[70]
175	20–2000	1	B	[71]
160, 170, 180, 190	2.5–758	0.5, 1, 2	B, M, R	[52]
190	20–1000	1	B	[63]
170, 175, 180, 190	40–160	/	/	[55]
180	20–1000	2	R	[53]
180	20–500	1, 1.5, 2, 2.5	B, M, R	[57]
180	20–500	1, 1.5, 2, 2.5	B, M, R	[58]
180	20–500	1, 1.5, 2, 2.5	B, M, R	[43]
180	20–1000	1, 1.5, 2	B, M, R	[59]
180	20–5000	1, 1.5, 2	B, M, R	[60]
180	20–5000	1, 1.5, 2	/	[65]
180	20–2000	1	B, M, R	[54]
180	20–2000	1, 1.5, 2	B, M	[61]
190	10–1000	2	/	[49]
215	20–5000	1	/	[48]
190	10–1000	2	/	[49]
170	3–1000	2	B, R	[13]
195	9–50	S 1.95, 2.5, 3.3, 4	M, R	[46]
185	50–90	S 1.33, 1.95, 4	M, R	[31]
195	5–100	S 1.95, 3.3, 4, 5.4	M, R	[35]
195	5–100	S 1.3, 1.95, 4	M, R	[37]
195	1–90	S 1.3, 2, 4,6	M, R	[36]
190	50–5000	2	B, M, R	[33]
190	10–2000	S 1, 1.5, 2	M, R	[33]
180	100–500	2	/	[73]
/	5–300	/	/	[64]
180	20–500	1, 1.5, 2	M	[67]
190	15–912	2	/	[56]
190	2–30	5	/	[66]
180	20–2000	1, 1.5, 2	B, M	[61]
240	50–1000	/	/	[68]
190, 195, 200, 205	0.3–10	4	/	[50]
175	50–2000	1	/	[51]

**Table 5:** Formulations tested with rotational rheometry.

Matrix	Wood Type	Wood wt.%	Coupling Agent	Lubricant	Other	Ref.
**HDPE**	Poplar	40	/	/	/	[69]
	Poplar	40	/	/	/	[70]
	Poplar	65	/	/	/	[71]
	Poplar	10, 20, 30, 40, 50, 60	4% MAPE	/	10–50% lignin	[84]
	Poplar	20, 30, 40, 50	5% MAPE	/	/	[79]
	Pine	35, 40	4% MAPE	5% ester	/	[53]
	Pine	14–33	2% MAPE	/	/	[85]
	Pine	10, 20, 30, 40, 50, 60, 70	0.5–5% silane	/	/	[96]
	Pine	30, 50	MAPE	Struktol	/	[107]
	Maple	30, 45, 60	1% MAPE	/	/	[60]
	Maple	55, 57, 60	2% MAPE	2% stearate 1% EBS ^1^ 2.7% OP100 ^2^	/	[58]
	Maple	25, 50, 60, 70	/	/	/	[54]
	Maple	5, 10, 20, 30, 50, 60	/	/	/	[65]
	Maple	30, 40	3% silane	/	1–5% mineral	[72]
	Maple	70	/	/	/	[78]
	Eucalyptus	50	7% MAPE	/	/	[55]
	Mangrove	10, 20, 30	/	/	/	[83]
	Spruce	20, 30, 40, 50, 60	3% MAPE	/	/	[93]
	Bamboo	10, 20, 30, 40, 50, 60	0.5% MAPE	7% Paraffin	1% Aox	[92]
	Aspen	/	3–9% GPE ^3^, GPW ^4^	/	/	[104]
**LDPE**	Poplar	40	3% MAPE	/	2% EPDM ^5^	[98]
	Birch	/	/	/	/	[99]
**PP**	Red pine	10, 20, 30	0.5–1% Silane	/	/	[90]
	Pine	20, 40, 60	4–16% MAPP	/	/	[77]
	Pine	30	/	/	/	[97]
	Pine	30	6–8% MAPP	1–2% Struktol	/	[101]
	Wood flour	40	5% MAPP	1–4% wax or SA ^6^ 2–4% stearate	/	[95]
	Wood flour	10, 20, 30, 40	2% MAPP	/	/	[94]
	White fir	30, 50, 70	/	/	/	[13]
	White fir	30, 50, 70	/	/	/	[89]
	Russian fir	50, 60, 70	/	/	/	[86]
	Bamboo	30, 40, 50	3,5% MAPP	/	/	[87]
	Lignocel	40, 60	2.5–10% comm.	/	/	[102]
**PP/HDPE**	Poplar	70	0.5,1,1.5,2 MAH ^7^	/	/	[103]
**TPU**	Poplar	10, 20, 30, 40	5% EPDM-g-MAH, POE ^8^-g-MAH, PEG6000 ^9^, Chitosan, MDI ^10^	/	/	[82]
**PLA**	Poplar	10, 20, 30, 40	/	/	/	[14]
	Spruce	10,30	/	/	/	[88]
	Spruce	10, 20, 30	/	/	/	[18]
**Mater-Bi ^11^**	Wood flour	15	/	/	/	[15]

^1^ Ethylene bis stearamide, ^2^ commercial lubricant, ^3^ grafted polyethylene, ^4^ grafted wax, ^5^ ethylene propylene diene monomer rubber, ^6^ stearic acid, ^7^ maleic anhydride, ^8^ polyolefin elastomer, ^9^ polyethyleneglycol 6000, ^10^ diphenylmethyl propane diisocyanate, ^11^ vegetable oil-derived polymer.

**Table 6:** Formulations tested with rotational rheometry.

Temperature (°C)	Frequency (rad s^−1^)	Strain (%)	Plates Diameter (mm)	Gap (mm)	Ref.
175	0.1–500	0.05	25	2	[69]
175	0.1–500	0.05	25	2	[70]
175	0.6–628	0.05	25	2	[71]
180	0.5–1000	/	25	/	[84]
170	0.01–100	0.1	25	1	[79]
180	0.1–100	0.1	25	2	[53]
190	0.06–628	/	25	1	[85]
170	0.01–10	/	25	2	[96]
170	0.1–100	/	25	1	[107]
180	0.1–100	/	/	/	[60]
180	0.01–100	0.1	25	2	[58]
180	0.1–100	0.1	25	1.5	[54]
180	0.1–100	0.1	25	1.5	[65]
200	0.6–628	/	25	1.5	[72]
180	0.01–100	0.01	25	2.5	[78]
210	0.1–500	/	25	2	[55]
170	0.05–500	0.05	25	0.5	[83]
170	0.6–629	/	25	/	[93]
170, 190	0.6–62.8	1	/	/	[92]
210	0.1–500	/	25	2	[104]
160	0.01–100	/	25	2	[98]
140, 160, 180	0.1–1000	3	25	1.5	[99]
200	0.01–60	/	25	1	[90]
180	0.1–100	0.03	25	/	[77]
200	0.1–100	5	25	1	[97]
190	0.01–100	0.2	25	1	[101]
190	0.06–628	0.1	25	2.5	[95]
190	0.1–200	1	25	2	[94]
170	0.1–100	0.01,0.02	25	2.5	[13]
170	0.1–100	0.01,0.02	25	2.5	[89]
175, 195	0.63–500	0.1,5	25	1,4	[86]
190	0.01–100	/	30	2.2	[87]
190	0.3–3962	6.28	35	1	[102]
190	0.1–100	/	25	2	[103]
200	0.06–628	/	25	3	[82]
175	0.06–628	0.1	25	2	[14]
155	0.1–99	0.05,0.1	25	2	[88]
155, 165, 175	0.1–100	0.05,0.1	25	2	[18]
140	/	/	/	/	[15]

**Table 7:** General results obtained varying the main parameters (viscosity η, complex viscosity $\eta^{*}$, storage modulus, $G^{\prime }$, loss modulus, $G^{\prime \prime }$) that affect the rheological properties. ↑ stands for “increase”, ↓ stands for “decrease”,/stands for “not present in the literature”, and ? stands for “still debated in the literature”.

	Pressure-Driven		Rotational		
Parameters	η	Wall Slip	$\eta^{*}$	$G^{\prime }$	$G^{\prime \prime }$
↑ Wood content	↑	↑	↑	↑	↑
↑ Particle size	↓	↑	↓	↓	↓
↑ Matrix molecular weight	↓	↓	/	/	/
↑ Testing temperature	↓	/	↓	↓	↓
Matrix recycling	/	/	↓	↓	↓
Coupling agents	?	/	?	?	?
Lubricants	/	↑	↓	↓	↓
Toughening agents	/	/	/	↑	/
